# *Lactobacillus rhamnosus* GR-1 alleviates *Escherichia coli*-triggered bovine endometrial epithelial cells damage via the reactive oxygen species-mitochondrial pathway

**DOI:** 10.5713/ab.25.0031

**Published:** 2025-06-04

**Authors:** Xiaowei Feng, Yan Li, Xiangfu Wen, Mingque Feng, Tianxiong Jin, Muhammad Shahid, Yan Sun, Jiawei Liu, Bei Liu, Jia Cheng, Mingchao Liu

**Affiliations:** 1 College of Veterinary Medicine, Hebei Agricultural University, Baoding, China; 2 Key Laboratory of Healthy Breeding in Dairy Cattle (Co-construction by Ministry and Province), Ministry of Agriculture and Rural Affairs, Hebei Agricultural University, Baoding, China; 3 Center of Microbiology and Biotechnology, Veterinary Research Institute, Peshawar, Pakistan

**Keywords:** Apoptosis, Bovine Endometritis, *Escherichia coli*, *Lactobacillus rhamnosus* GR-1, Reactive Oxygen Species

## Abstract

**Objective:**

The objective of this study was to evaluate the function of the reactive oxygen species (ROS)-mitochondrial pathway in attenuating *Escherichia coli* (*E. coli*) induced apoptosis in bovine endometrial epithelial cells (BENDs) by *Lactobacillus rhamnosus* (*L. rhamnosus*) GR-1.

**Methods:**

The BENDs were exposed to preincubation with and without *L. rhamnosus* GR-1 for 3 hours (h) and they were later subject to *E. coli* for 6 h. The release of lactate dehydrogenase (LDH), the expression of oxidative factors, adhesion and invasion of *E. coli*, the expression of mitochondrial membrane potential (MMP), apoptotic rate and apoptosis-associated protein expression were observed. Then, ROS expression, MMP level and cell rate apoptosis rate were further detected after the intervention of antioxidant n-acetyl-l-cysteine (NAC).

**Results:**

*L. rhamnosus* GR-1 was capable of obviously alleviating the content of LDH, ROS expression, *E. coli* adhesion and invasion, the apoptotic rate and MDA concentrations in BENDs induced by *E. coli* (p<0.01). In addition, *L. rhamnosus* GR-1 could notably promote the levels of antioxidant factors (SOD, GSH, T-AOC) (p<0.01), inhibit the depolarization of MMP (p<0.01), and levels of apoptosis-related factors (*Bax*, *Caspase-3* and *Cyt-c*), and promote anti-apoptosis factor (*Bcl-2*) (p<0.01). Moreover, after the intervention of antioxidant NAC, ROS expression, MMP depolarization, and apoptotic rate induced by *E. coli* in BENDs were decreased (p<0.01).

**Conclusion:**

In *E. coli*-induced apoptosis in BENDs, therapeutic strategies aimed at down-regulating ROS and MDA and up-regulating antioxidant factors may maintain mitochondrial function and provide theoretical support for the use of probiotics in bovine endometritis.

## INTRODUCTION

Bovine endometritis is a postpartum uterine inflammation of dairy cows, resulting in infertility, reduced milk production, and increased treatment costs, causing significantly economic losses [1,2]. *Escherichia coli* is one of the main pathogens leading to bovine endometritis [3]. Antibiotics have commonly been adopted for treating bovine endometritis, but due to antibiotic overuse, the problem of antibiotic resistance is becoming more serious, also posing a tremendous threat to dairy health [4]. Hence, it is critical to elucidate the underlying mechanisms of endometritis and to identify alternative treatments to prevent and manage bovine endometritis.

*E. coli* infection can be recognized by receptors expressed in bovine endometrial epithelial cells (BENDs), triggering an inflammatory response [5]. The progression of several inflammatory diseases has been linked to reactive oxygen species (ROS). Studies have shown that *E. coli* significantly elevates ROS levels, suppresses antioxidant enzyme expression, enhances lipid peroxidation, and ultimately triggers oxidative stress [6]. It is generally accepted that elevated ROS levels contribute to abnormalities in mitochondrial membrane potential (MMP) [7]. Then, pro-apoptotic proteins Cytochrome c (Cyt-c) is capable of releasing from mitochondria to the cytoplasm, with recruitment as well as the activation of procaspase-9. Next, the cleaved caspase-9 enzyme is found to activate the executioner procaspase-3, leading to the induction of apoptosis through the formation of cleaved caspase-3 [8]. Therefore, inhibition of ROS-mediated apoptosis may be an effective treatment for inflammatory diseases such as bovine endometritis.

As the alternative to antibiotics, probiotics have attracted particular attention. They are referred to as “living microorganisms that, when given in sufficient quantities, delivers host health advantages” [9]. Probiotics are widely used to address animal diseases resulting from bacterial infections. There are many kinds of probiotics widely used in endometritis, including *Lactobacillus*, *Bifidobacterium* and *Streptococcus* etc. [10,11]. *Lactobacillus* has anti-inflammatory, antioxidant, anti-apoptotic and antibacterial properties [12,13]. Previous studies have found that *L. plantarum* KSFY06 reduces liver injury by exerting antioxidant and anti-inflammatory effects [14], and *Lactobacillus rhamnosus* ameliorates BENDs inflammatory response as well as apoptosis caused by *B. cereus* [15]. Meanwhile, according to our previous research, *L. rhamnosus* GR-1 has demonstrated the ability to reduce apoptosis in the BENDs infected with *E. coli* [16]. However, the mechanism of *L. rhamnosus* GR-1 in attenuating the apoptosis of BENDs triggered by *E. coli* still needs to be clarified.

Therefore, we hypothesized that *L. rhamnosus* GR-1 had the capability of hindering the apoptosis of BENDs through inhibiting the mitochondrial apoptosis pathway and speculated that ROS exerted a vital role during this process. The current work was to investigate the inhibitory mechanistic function of *L. rhamnosus* GR-1 on the pathogenic effect in BENDs triggered by *E. coli* and to provide new insights for *L. rhamnosus* GR-1 to mitigate *E. coli*-induced injury in BENDs.

## MATERIALS AND METHODS

### Cell and bacterial strains

BENDs (BNCC340413, BNCC, Beijing, China) were incubated in 89% DMEM/F12 (Procell, Wuhan, China) supplemented with 10% concentration of fetal bovine serum (MeliunBio, Dalian, China), and 1% concentration of penicillin/streptomycin (Solarbio, Beijing, China)_,_ and passaged at a density of 1×10^6^/well in 6-well plates for subsequent experiments.

*L. rhamnosus* GR-1 strain (ATCC 55826) was provided by the Laboratory of Clinical Nutrition and Immunology of the College of Veterinary Medicine of the China Agricultural University. The *L. rhamnosus* GR-1 was grown in MRS medium (AOBOX, Beijing, China) at 37°C and it was diluted in DMEM/F12 basic medium (Procell) to a density of 5×10^6^ CFU/mL for follow-up tests.

*E. coli* strain O111: K58 (CVCC1450) was provided by the Laboratory of Clinical Nutrition and Immunology of the College of Veterinary Medicine of the China Agricultural University. The *E. coli* was grown in LB broth (AOBOX) with 180 rpm at 37°C and it was diluted in DMEM/F12 basic medium (Procell) to a density of 5×10^5^ CFU/mL for infecting the cells.

### Cell treatment in different experiments

BENDs were seeded in a 6-well plate at a density of 1×10^6^ and incubated at 37°C for 24 h to reach 90%–100% density, then washed twice with PBS. BENDs were pretreated with *L. rhamnosus* GR-1 (5×10^6^ CFU/mL) for 3 h and then infected with *E. coli* (5×10^5^ CFU/mL) for 6 h [5]. After treatment, cell samples were collected for subsequent experimental analysis.

BENDs were inoculated into 6-well plates at a density of 1×10^6^ cells/well and incubated at 37°C for 24 h to reach 90%–100% density, then washed twice with PBS. After ROS scavenger NAC (10 μM) pretreatment for 3 h. Subsequently, the cells were co-incubated with *E. coli* for 6 h in order to determine the effect of ROS deletion on the oxidative stress and apoptotic response of BENDs. After treatment, cell samples were collected for subsequent experimental analysis.

### Cytotoxic lactate dehydrogenase release assay

BENDs were added 5×10^6^ CFU/mL of *L. rhamnosus* GR-1 for 3 h, followed by the action of 5×10^5^ CFU/mL of *E. coli* for 6 h. The cytotoxic effects of *L. rhamnosus* GR-1 on *E. coli*-infected BENDs were assessed using a lactate dehydrogenase (LDH) assay kit (Jiancheng Bioengineering Institute, Nanjing, China). The supernatant was collected according to manufacturer’s instructions. The optical density was measured at 450 nm with a Microplate Spectrophotometer (Bio Tek, Winooski, VT, USA).

### Adhesion and invasion

Adhesion and invasion were measured following the method described by Wu [17]. Briefly, BENDs were pretreated with *L. rhamnosus* GR-1 for 3 h and then exposed to *E. coli* treatment for 6 h. Cells were washed three times with PBS to remove non-adherent bacteria and then harvested by treatment with 0.25% trypsin for 3 min at 37°C. The cells were then incubated with DMEM/F12 medium to determine the number of bacteria adhering to the surface of BENDs. Cell suspensions were diluted with DMEM/F12 medium and then incubated on EMB Agar plates to determine the number of bacteria attached to the surface of BENDs.

To evaluate the number of *E. coli* invading BENDs, the cells were washed with PBS and treated with 1 mL of DMEM/F12 containing Gentamicin (100 μg/mL) for 2 h. After that, the cells were lysed with 0.5% Triton X-100. The lysate was diluted and cultured on EMB Agar plate plate as described above. The colonies were counted, and the invasion rate of *E. coli* into BENDs was calculated.

### Measurement of the levels of intracellular reactive oxygen species

Intracellular levels of ROS were measured using 2,7-dichlorofluorescein diacetate (DCFH-DA) (Beyotime, Beijing, China). After treating the cells, 1 mL of 10 μmol/L DCFH-DA was added and incubated at 37°C for 20 min. The cells were then washed three times with a medium devoid of FBS and and observed under a fluorescence microscope (IX51; Olympus, Tokyo, Japan).

### Detection of oxidation-related factor

Oxidation-related factors including SOD, GSH, T-AOC and MDA were assessed in the cells using a kit (Jiancheng Bioengineering Institute, Nanjing, China). After modeling was completed, cell suspensions were collected according to the manufacturer’s instructions and then centrifuged at 1,000 r/min for 10 min. The optical density was measured with a Microplate Spectrophotometer (synergyLX, Bio Tek).

### Assessment of mitochondrial membrane potential (ΔΨm)

The MMP of different groups of cells were assessed using the Enhanced Mitochondrial MMP Detection Kit (JC-1) (Beyotime). The degree of mitochondrial depolarization was assessed by calculating the relative ratio of red (JC-1 aggregate) to green (JC-1 monomer) fluorescence. After completing the modeling, 2 mL of JC-1 staining working solution was added to each group, and subsequently, the cells were rinsed twice with 2 mL of JC-1 staining buffer, and, finally, 2 mL of complete DMEM/F12 medium was added and the cells were visualized using fluorescence microscopy (IX51; Olympus).

### Measurement of cell apoptosis

The apoptosis rate of BENDs were assessed using the Annexin V-FITC/PI Apoptosis Detection Kit (4ABioteece, Beijing, China). Cells were collected by adding 1× binding buffer and 5 μL of Annexin V/FITC, followed by a 5 min incubation at room temperature away from light. Then, 10 μL of 20 μg/mL propidium iodide solution and 400 μL of PBS were added. Finally, the samples were analysed using a flow cytometer (BD, Franklin Lakes, NJ, USA).

### Real-time quantitative polymerase chain reaction

The total RNA was extracted from BENDs using Triquick Reagent (Solarbio). The RNA quality of all samples was assessed by NanoDrop ND-2000 spectrophotometer. The RevertAid First Strand cDNA Synthesis Kit was used to synthesize first-strand cDNA (Tiangen, Beijing, China). Primer-BLAST software was used to design primers (Table 1). The real-time polymerase chain reaction (RT–PCR) was conducted on a LightCycler 480 system using SuperReal PreMix Plus (Tiangen). The reaction conditions were set at 95°C for 15 min, followed by 40 cycles consisting of denaturation at 95°C for 10 s and annealing/elongation at 60°C for 30 s. Moreover, relative transcript expression levels were calculated via the 2^−ΔΔCT^.

### Western blot

The total protein was extracted from cells using a whole protein extraction kit (Solarbio). Determination of group protein concentrations using the BCA Protein Concentration Assay Kit (Solarbio). Subsequently, proteins were separated by SDS-PAGE on a 12.5% gel and transferred to a PVDF membrane. The membrane was blocked with 5% skimmed milk for 60 min at 37°C, and then incubated overnight at 4°C with primary antibodies, including rabbit anti-Bcl-2 (1:500), rabbit anti-Bax (1:500), rabbit anti-caspase-3 (1:500), mouse β-actin (1:1,000) (Bioss, Beijing, China), and rabbit anti-Cyt-c (1:1,000) (Cell Signaling Technology, Danvers, MA, USA). After this, the membrane was incubated with secondary antibodies at 37°C for 1 h: goat anti-rabbit IgG (Bioss) and goat anti-mouse IgG (1:1,000) (Proteintech, Wuhan, China), followed by ultrasensitive ECL chemiluminescence development. The relative intensity of each band was assessed by Image J 1.47 v software.

### Statistical analyses

The experimental results were derived from three independent experiments, each performed in triplicate. The experimental unit of investigation for each variable was a cell well. Values were expressed as means±standard errors of means (SEMs). Statistical significance was calculated using one-way ANOVA, followed by the Duncan and LSD multiple tests, using SPSS 27.0 (SPSS, Chicago, IL, USA), A p-value<0.05 was considered statistically significant. GraphPad Prism 8.0 software was used for all analyses.

## RESULTS

### Effect of *Lactobacillus rhamnosus* GR-1 on the activity of bovine endometrial epithelial cells infected with *Escherichia coli*

**Figure 1:**
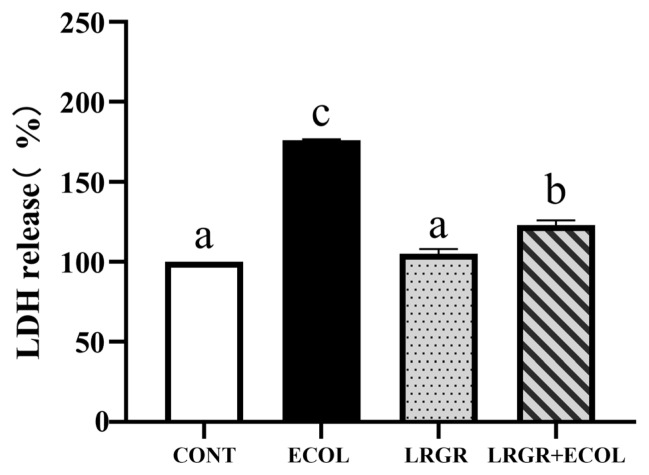
Effect of *Lactobacillus rhamnosus* GR-1 on the activity of BENDs infected with *Escherichia coli*. BENDs (90%–100%) were treated with *L. rhamnosus* GR-1 (5×10^6^ CFU/mL), *E. coli* (5×10^5^ CFU/mL) alone or a combination of *L. rhamnosus* GR-1 (5×10^6^ CFU/mL), *E. coli* (5×10^5^ CFU/mL) for 9 h. Cell activity was assessed by assaying the LDH release rate, using a Microplate Spectrophotometer to measure absorbance at 450 nm. The LDH release rate was calculated and expressed as a percentage of cells in CONT group (received no *L. rhamnosus* GR-1 and *E. coli*). Data represented are means± standard errors of means (SEMs) from triplicate experiments. ^a–c^ If the same letter appears on the bars, the difference is not significant (p>0.05). If there is no common letter among the bars, the difference is significant (p<0.05). LDH, lactate dehydrogenase; BENDs, bovine endometrial epithelial cells.

The effect of *L. rhamnosus* GR-1 on the rate of LDH release in *E. coli* infected BENDs is shown in Figure 1, where the rate of LDH release was significantly higher in the *E. coli* infected group (p<0.01), while pretreatment with *L. rhamnosus* GR-1 significantly reduced the rate of LDH release (p<0.01).

### Effect of *Lactobacillus rhamnosus* GR-1 pretreatment on adhesion and invasion of bovine endometrial epithelial cells by *Escherichia coli*

**Figure 2:**
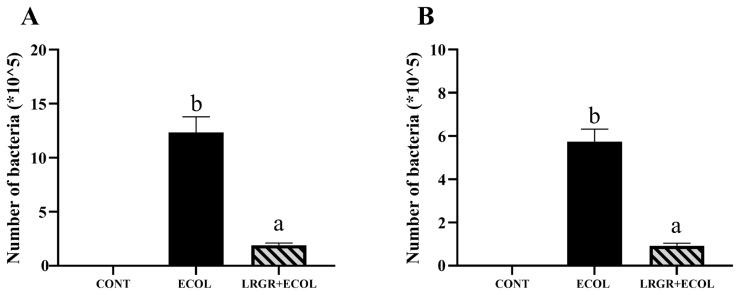
Effect of *Lactobacillus rhamnosus* GR-1 on the activity of BENDs infected with *Escherichia coli*. BENDs (90%–100%) were treated with *E. coli* (5×10^5^ CFU/mL) alone or a combination of *L. rhamnosus* GR-1 (5×10^6^ CFU/mL), *E. coli* (5×10^5^ CFU/mL) for 9 h. (A) Adhesion of *E. coli*-infected bMECs in pretreatment of *L. rhamnosus* GR-1. (B) invasion of *E. coli*-infected bMECs in pretreatment of *L. rhamnosus* GR-1. Data represented are means±standard errors of means (SEMs) from triplicate experiments. ^a,b^ If there is no common letter among the bars, the difference is significant (p<0.05). BENDs, bovine endometrial epithelial cells.

The results of the effect of *L. rhamnosus* GR-1 pretreatment on the adhesion and invasion of BENDs by *E. coli* are shown in Figure 2. Pretreatment with *L. rhamnosus* GR-1 could significantly reduce the adhesion and invasion of BENDs by *E. coli* (p<0.01).

### Effect of *Lactobacillus rhamnosus* GR-1 on oxidative stress induced by *Escherichia coli* in bovine endometrial epithelial cells

**Figure 3:**
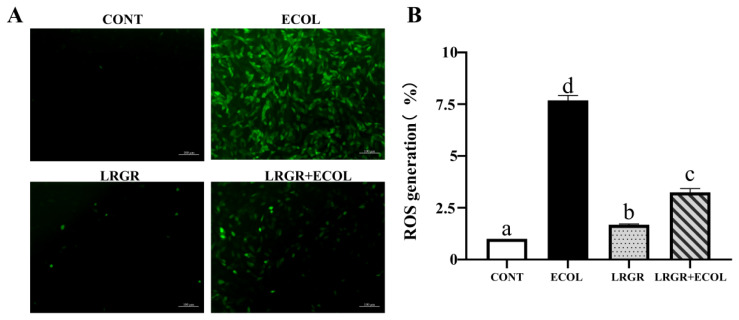
Effects of *Lactobacillus rhamnosus* GR-1 on ROS levels in *Escherichia coli*-treated BENDs. BENDs (90%–100%) were treated with *L. rhamnosus* GR-1 (5×10^6^ CFU/mL), *E. coli* (5×10^5^ CFU/mL) alone or a combination of *L. rhamnosus* GR-1 (5×10^6^ CFU/mL), *E. coli* (5×10^5^ CFU/mL) for 9 h. (A) Representative micrographs show DCF fluorescence of control cells, cells treated with *L. rhamnosus* GR-1 or *E. coli* alone, and cells co-treated with *L. rhamnosus* GR-1 and E. coli. (B) Quantification of DCF fluorescence was based on the images in Figure 3A. ROS generation was calculated and expressed as a percentage of fluorescence intensity for the CONT group (not receiving *L. rhamnosus* GR-1 and *E. coli*). Data represented are means±standard errors of means (SEMs) from triplicate experiments. ^a–d^ If there is no common letter among the bars, the difference is significant (p<0.05). ROS, reactive oxygen species; BENDs, bovine endometrial epithelial cells; DCF, dichlorofluorescein.

The results of the effect of *L. rhamnose* GR-1 on ROS levels in *E. coli*-infected BENDs are shown in Figure 3. The results showed a highly significant increase in cellular ROS expression in the *E. coli* infection group (p<0.01). Whereas, *L. rhamnosus* pretreatment reduced ROS expression extremely significantly (p<0.01) (Figure 3).

**Figure 4:**
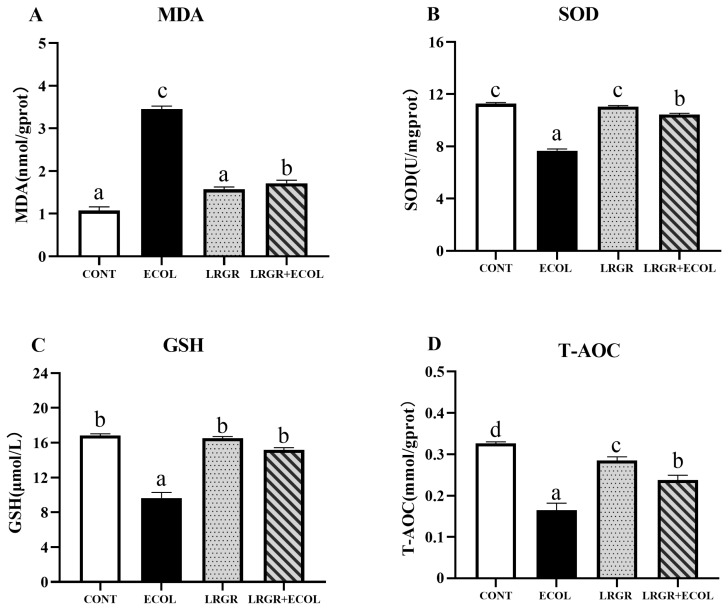
Effects of *Lactobacillus rhamnosus* GR-1 on oxidative factors levels in *Escherichia coli*-treated BENDs. BENDs (90%–100%) were treated with *L. rhamnosus* GR-1 (5×10^6^ CFU/mL), *E. coli* (5×10^5^ CFU/mL) alone or a combination of *L. rhamnosus* GR-1 (5×10^6^ CFU/mL), *E. coli* (5×10^5^ CFU/mL) for 9 h. (A) MDA content. (B) SOD content. (C) GSH content. (D) T-AOC content. Absorbance was measured using a Microplate Spectrophotometer and content was calculated. Data represented are means±standard errors of means (SEMs) from triplicate experiments. ^a–d^ If the same letter appears on the bars, the difference is not significant (p>0.05). If there is no common letter among the bars, the difference is significant (p<0.05). MDA, malondialdehyde; SOD, superoxide dismutase; GSH, glutathione; T-AOC, total antioxidant capacity; BENDs, bovine endometrial epithelial cells.

The results of the effect of *L. rhamnosus* GR-1 on the expression of oxidation-related factors in *E. coli* infected BENDs are shown in Figure 4. The *L. rhamnosus* pretreatment significantly repressed the *E. coli*-induced activity of oxidation factors (MDA), while the activity of antioxidant (SOD, GSH and T-AOC) was significantly increased (p<0.01).

### Effect of *Lactobacillus rhamnosus* GR-1 on mitochondrial membrane potential of bovine endometrial epithelial cells induced by *Escherichia coli*

**Figure 5:**
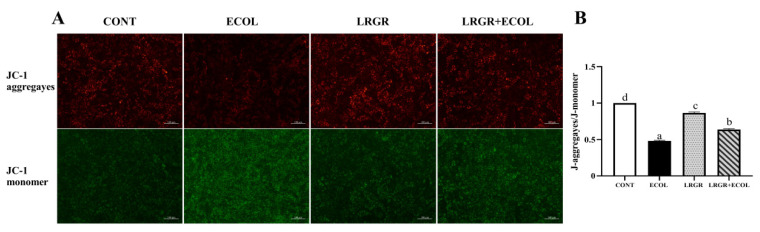
Effects of *Lactobacillus rhamnosus* GR-1 on MMP levels in *Escherichia coli*-treated BENDs. BENDs (90%–100%) were treated with *L. rhamnosus* GR-1 (5×10^6^ CFU/mL), *E. coli* (5×10^5^ CFU/mL) alone or a combination of *L. rhamnosus* GR-1 (5×10^6^ CFU/mL), *E. coli* (5×10^5^ CFU/mL) for 9 h. (A) Representative micrographs show JC-1 fluorescence of control cells, cells treated with *L. rhamnosus* GR-1 or *E. coli* alone, and cells co-treated with *L. rhamnosus* GR-1 and *E. coli*. (B) Quantification of JC-1 fluorescence was based on the images in Figure 5A. J-aggregayes/J-monomer was calculated and expressed as a percentage of fluorescence intensity for the CONT group (not receiving *L. rhamnosus* GR-1 and *E. coli*). Data represented are means±standard errors of means (SEMs) from triplicate experiments. ^a–d^ If there is no common letter among the bars, the difference is significant (p<0.05). MMP, mitochondrial membrane potential; BENDs, bovine endometrial epithelial cells.

The results of the effect of *L. rhamnosus* GR-1 on MMP levels in *E. coli*-infected BENDs are shown in Figure 5. The results showed that MMP levels were highly significantly lower (p< 0.01) in the cells of the *E. coli* infected group. Whereas, MMP levels were highly significantly elevated (p<0.01) in the cells of *L. rhamnosus* pretreated group.

### Effect of *Lactobacillus rhamnosus* GR-1 on apoptosis of bovine endometrial epithelial cells induced by *Escherichia coli*

**Figure 6:**
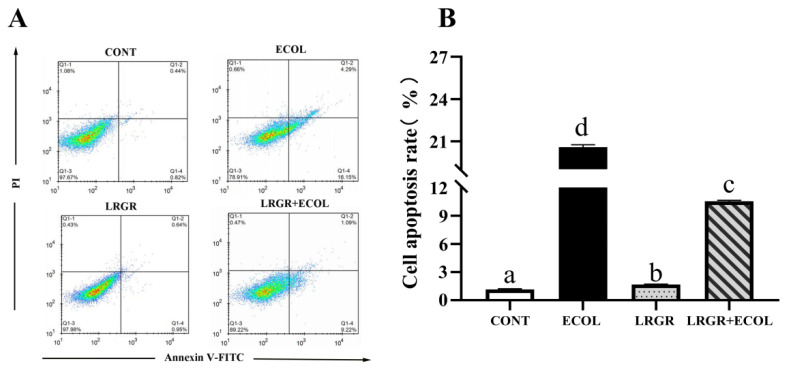
Effects of *Lactobacillus rhamnosus* GR-1 on apoptosis in *Escherichia coli*-treated BENDs. BENDs (90%–100%) were treated with *L. rhamnosus* GR-1 (5×10^6^ CFU/mL), E. coli (5×10^5^ CFU/mL) alone or a combination of *L. rhamnosus* GR-1 (5×10^6^ CFU/mL), *E. coli* (5×10^5^ CFU/mL) for 9 h. (A) Determination of apoptosis rates using flow cytometry. (B) Quantitative analysis of apoptosis rates was based on the images in Figure 6A. Cell apoptosis rate was calculated and expressed as a percentage of fluorescence intensity for the CONT group (not receiving *L. rhamnosus* GR-1 and *E. coli*). Data represented are means±standard errors of means (SEMs) from triplicate experiments. ^a–d^ If there is no common letter among the bars, the difference is significant (p<0.05). BENDs, bovine endometrial epithelial cells.

The results of the effect of *L. rhamnosus* GR-1 on apoptosis in *E. coli*-infected BENDs are shown in Figure 6. The results showed that the apoptosis rate was highly significantly increased (p<0.01) in the *E. coli* infected group, whereas *L. rhamnosus* GR-1 pretreatment resulted in a highly significant decrease in the apoptosis rate (p<0.01).

**Figure 7:**
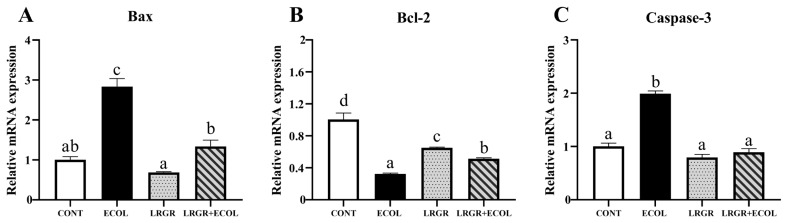
Effects of *Lactobacillus rhamnosus* GR-1 on the mRNA levels of Bcl-2, Bax and Caspase-3 in *Escherichia coli*-treated BENDs. BENDs (90%–100%) were treated with *L. rhamnosus* GR-1 (5×10^6^ CFU/mL), *E. coli* (5×10^5^ CFU/mL) alone or a combination of *L. rhamnosus* GR-1 (5×10^6^ CFU/mL), *E. coli* (5×10^5^ CFU/mL) for 9 h. (A) mRNA levels of the Bax were assessed by RT-qPCR. Relative transcript expression was calculated using the 2^−ΔΔCt^ method and presented as values relative to the CONT group (not receiving *L. rhamnosus* GR-1 and *E. coli*). (B) mRNA levels of the Bcl-2 were assessed by RT-qPCR. Relative transcript expression was calculated using the 2^−ΔΔCt^ method and presented as values relative to the CONT group (not receiving *L. rhamnosus* GR-1 and *E. coli*). (C) mRNA levels of the Caspase-3 were assessed by RT-qPCR. Relative transcript expression was calculated using the 2^−ΔΔCt^ method and presented as values relative to the CONT group (not receiving *L. rhamnosus* GR-1 and *E. coli*). Data represented are means±standard errors of means (SEMs) from triplicate experiments. ^a–d^ If the same letter appears on the bars, the difference is not significant (p>0.05). If there is no common letter among the bars, the difference is significant (p<0.05). Bax, Bcl-2-associated X protein; Bcl-2, B-cell lymphoma-2; BENDs, bovine endometrial epithelial cells; RT-qPCR, real-time quantitative polymerase chain reaction.

**Figure 8:**
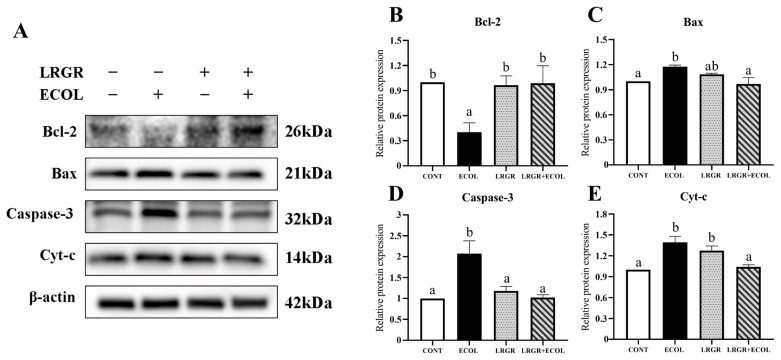
Effects of *Lactobacillus rhamnosus* GR-1on the expression of mitochondrial-mediated apoptosis pathway protein in *Escherichia coli*-treated BENDs. BENDs (90%–100%) were treated with *L. rhamnosus* GR-1 (5×10^6^ CFU/mL), *E. coli* (5×10^5^ CFU/mL) alone or a combination of *L. rhamnosus* GR-1 (5×10^6^ CFU/mL), *E. coli* (5×10^5^ CFU/mL) for 9 h. (A) Bcl-2, Bax, Caspase-3 and Cyt-c and β-actin (internal control) protein levels were detected using Western blot. (B) Quantification of Bcl-2 expression of three independent experiments and normalized to β-actin and represented in the bar graph. (C) Quantification of Bax expression of three independent experiments and normalized to β-actin and represented in the bar graph. (D) Quantification of Caspase-3 expression of three independent experiments and normalized to β-actin and represented in the bar graph. (E) Quantification of Cyt-c expression of three independent experiments and normalized to β-actin and represented in the bar graph. Data represented are means±standard errors of means (SEMs) from triplicate experiments. ^a,b^ If the same letter appears on the bars, the difference is not significant (p>0.05). If there is no common letter among the bars, the difference is significant (p<0.05). BENDs, bovine endometrial epithelial cells.

Subsequently, mRNA and protein expression levels of mitochondria-dependent apoptosis-related factors were further analyzed. The results are shown in Figures 7, 8. mRNA and protein levels of Bax and Caspase-3 were extremely significantly increased and Cyt-c protein expression was extremely significantly increased in *E. coli* infected group of cells (p< 0.01), whereas the mRNA and protein levels of Bax and Caspase-3 were significantly decreased after *L. rhamnosus GR-1* pretreatment (p<0.05), Cyt-c protein expression was highly significantly reduced (p<0.01). mRNA and protein expression of Bcl-2 was highly significantly decreased (p<0.01) in *E. coli* infected group and increased (p<0.05) after *L. rhamnosus GR-1* pretreatment.

### Effect of reactive oxygen species signaling in *Lactobacillus rhamnosus* GR-1 on alleviating *Escherichia coli* induced-apoptosis in bovine endometrial epithelial cells

**Figure 9:**
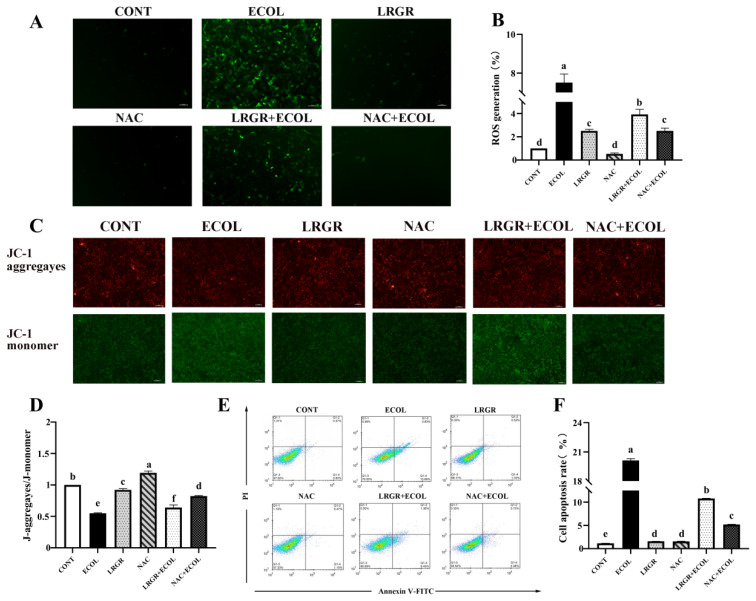
*Lactobacillus rhamnosus* GR-1 alleviates ROS-dependent apoptosis of BENDs induced by *Escherichia coli*. BENDs (90%–100%) were treated with *L. rhamnosus* GR-1 (5×10^6^ CFU/mL), *E. coli* (5×10^5^ CFU/mL), NAC (10 μM) alone or a combination of *L. rhamnosus* GR-1 (5×10^6^ CFU/mL) and *E. coli* (5×10^5^ CFU/mL), NAC (10 μM) and *E. coli* (5×10^5^ CFU/mL) for 9 h. (A) Representative micrographs show DCF fluorescence of control cells, cells treated with *L. rhamnosus* GR-1 or *E. coli* alone, and cells co-treated with *L. rhamnosus* GR-1 and *E. coli*. (B) Quantification of DCF fluorescence was based on the images in Figure 9A. ROS generation was calculated and expressed as a percentage of fluorescence intensity for the CONT group (not receiving *L. rhamnosus* GR-1 and *E. coli*). (C) Representative micrographs show JC-1 fluorescence of control cells, cells treated with *L. rhamnosus* GR-1 or *E. coli* alone, and cells co-treated with *L. rhamnosus* GR-1 and *E. coli*. (D) Quantification of JC-1 fluorescence was based on the images in Figure 9C. J-aggregayes/J-monomer was calculated and expressed as a percentage of fluorescence intensity for the CONT group (not receiving *L. rhamnosus* GR-1 and *E. coli*). (E) Determination of apoptosis rates using flow cytometry. (F) Quantitative analysis of apoptosis rates was based on the images in Figure 9E. Cell apoptosis rate was calculated and expressed as a percentage of fluorescence intensity for the CONT group (not receiving *L. rhamnosus* GR-1 and *E. coli*). Data represented are means±standard errors of means (SEMs) from triplicate experiments. ^a–f^ If the same letter appears on the bars, the difference is not significant (p>0.05). If there is no common letter among the bars, the difference is significant (p<0.05). ROS, reactive oxygen species; BENDs, bovine endometrial epithelial cells; DCF, dichlorofluorescein.

The results of the effect of NAC on alleviating *E. coli*-induced apoptosis in BEND cells are shown in Figure 9. The results showed that ROS level, MMP depolarization level and apoptosis rate were significantly reduced (p<0.05) in the *E. coli* group reinfected after NAC pretreatment.

## DISCUSSION

Bovine endometritis is a disease caused by postpartum bacterial infection. *E. coli* is one of the main pathogens which causes serious economic losses [18]. Our previous study found that *L. rhamnosus* GR-1 inhibited the inflammatory damage via NF-κB/MAPK pathway of bovine endometritis epithelial cells induced by *E. coli* [5]. Additionally, *L. rhamnosus* GR-1 has an anti-apoptotic effect in bovine endometritis epithelial cells induced by *E. coli* [16], but its molecular mechanism is unclear. In the current study, we found *L. rhamnosus* GR-1 improved the antioxidant effect in *E. coli*-infected BENDs. Meanwhile, ROS-mitochondrial pathway plays an important role in anti-apoptotic effects of *L.rhamnosus* GR-1 in *E. coli*-infected BENDs.

LDH is a stable intracellular cytoplasmic enzyme that is rapidly released into the cell culture supernatant when the cytoplasmic membrane is disrupted, which is a major feature of cells undergoing apoptosis or other forms of cellular damage [19]. LDH release assays are often used as complementary assays to assess cell membrane integrity [20]. Our study found that *L. rhamnosus* GR-1 alone did not cause cellular damage, while pretreatment with *L. rhamnosus* GR-1 attenuated *E. coli*-induced LDH release.

Bacterial adhesion to host epithelial cells is a critical step in initiating infection [21]. In contrast, the surface proteins and exopolysaccharides of probiotics can inhibit the formation of pathogenic biofilms by inhibiting adhesion, thus playing an important role in promoting pre-colonization and repelling pathogenic bacteria [22]. In the present study, *L. rhamnosus* GR-1 were able to significantly reduce the adhesion and colonization of *E. coli*, a result that is consistent with other studies, suggesting that *L. rhamnosus* GR-1 are effective in inhibiting the adhesion and invasion of *E. coli* [17], which may be due to the inhibition of adhesion and the blocking of the binding site of the pathogen to the cell-surface receptor to prevent formation of pathogenic biofilm.

Excess ROS lead to increased lipid peroxidation products and decreased antioxidant indices [23], the imbalance between the oxidation system and the antioxidant systems is an important factor leading to oxidative stress in dairy cows. After invasion of *E. coli* to the endometrium, *E. coli* can stimulate cells to cause oxidative stress in the cells [24]. In this study, we confirmed that *E. coli* could elevate ROS levels, increased the expression of MDA, and suppressed the expression of SOD, GSH and T-AOC, consistent with previous finding [25]. Lactic acid bacteria can regulate the expression of antioxidant enzymes, thereby alleviating oxidative stress [26,27]. In this study, *L. rhamnosus* GR-1 effectively reduced ROS levels. Subsequently, we observed that *L. rhamnosus* GR-1 significantly inhibited MDA activity while enhancing the activity of SOD, GSH and T-AOC. Therefore, *L. rhamnosus* GR-1 can effectively inhibit the oxidative damage of cells caused by *E. coli*.

Mitochondria are the energy center of the cell and are critical to cell survival. Studies have shown that excessive production of ROS can impair mitochondrial function, cause increased mitochondrial membrane permeability, and disrupt membrane integrity, leading to a decrease in MMP [28]. Song et al [29] found that excess ROS induced mitochondrial damage and decreased MMP levels in BEECs. Li et al [30] also found that *E. coli* could cause mitochondrial damage in MAC-T cells, and *L. rhamnosus* GR-1 could attenuate *E. coli*-induced mitochondrial damage. In agreement with our results of evaluating MMP levels in BENDs, *E. coli* infection significantly decreased MMP levels, and MMP levels in BENDs were significantly reduced with *L. rhamnosus* GR-1 Pretreatment significantly increased MMP levels in BENDs.

Apoptosis plays an important role in cellular physiological processes. Studies have shown that ROS overproduction leads to apoptosis and induces mitochondria-mediated production of endogenous apoptosis-associated protein Bax [31], which leads to the release of Cyt-c from the mitochondria to activate Caspase-3 leading to apoptosis [32]. In the present study, *L. rhamnosus* GR-1 significantly attenuated *E. coli*-induced apoptosis by decreasing Bax, Caspase-3 and Cyt-c expression and increasing Bcl-2 expression. Zhang et al [33] found that ROS overproduction induced apoptosis with an increase in the BAX to BCL-2 ratio and an increase in Caspase-3 content. Zheng et al [34] found that *L. rhamnosus* CY12 could alleviate LPS-induced apoptosis, and Zhang et al [35] found that *L. plantarum* Lp2 inhibited the mitochondria-mediated apoptosis pathway. Our data further confirm these studies and reinforce the important role of probiotics in regulating apoptosis.

ROS is produced by xenobiotic exposure and physiological metabolism, which can induce cell apoptosis [36,37]. ROS function as “redox messengers” involved in intracellular modulation and signal transduction, whereas elevated levels of ROS lead to oxidative modification of cellular macromolecules, suppression of protein activities, and promotion of cell apoptosis [38]. Mechanisms of ROS-induced apoptosis include caspase activation and mitochondrial damage among others [39].

In light of this, we hypothesized that ROS are required for *E. coli* to induce mitochondrial damage and apoptosis. Therefore, we therefore explored the importance of ROS for *E. coli*-induced mitochondrial damage and apoptosis by treating cells with NAC, a ROS scavenger. The results showed that NAC significantly reduced ROS expression and apoptosis and increased MMP levels, which is consistent with previous findings [40]. The observations suggest that ROS release is required for *E. coli*-induced mitochondrial damage. Meanwhile, *L. rhamnosus* GR-1 could attenuate *E. coli*-induced ROS activation and apoptosis. Therefore, we hypothesized that *L. rhamnosus* GR-1 has the ability to scavenge ROS. Together, our findings suggest that ROS-mediated mitochondrial apoptosis is one of the pathways by which *L. rhamnosus* GR-1 protects cells from *E. coli*-induced cellular injury.

In this study, only BEND was used as an *in vitro* infection model to explore the protective effects of *L. rhamnosus* GR-1 against endometritis in dairy cows, therefore, we must emphasize that this is only a preliminary exploration of the protective effects of *L. rhamnosus* GR-1 through the ROS-mediated mitochondrial pathway. Future studies need to establish *in vivo* infection models to further elucidate the protective effects of *L. rhamnosus* GR-1 in animals.

## CONCLUSION

In conclusion, our findings suggest that *L. rhamnosus* GR-1 pretreatment attenuates *E. coli*-induced cellular damage, in part by inhibiting the damaging effects of *E. coli* by inhibiting its adhesion and invasion with BENDs and by modulating the ROS-mediated mitochondria-dependent apoptotic pathway. These findings deepen the understanding of probiotic immunoprotection and contribute to its application in the prevention and treatment of endometritis in dairy cows. However, studies on the *in vivo* protective mechanisms and probiotic routes of administration are necessary.

## Figures and Tables

**Table 1:** The primer sequences, amplified fragment length, and sequence number of four target genes used in this study

Target genes	Primer sequence (5′–3′)		Product length (bp)	Serial number
*Bcl-2*	F	AGGGGTCATGTGTGTGGAGAGC	99	NM_001166486.1
	R	GTGTGCAGGTGCCGGTTCAG		
*Bax*	F	CGGCCTCCTCTCCTACTTTGGG	83	NM_173894.1
	R	TGGTGAGCGAGGCGGTGAG		
*Caspase-3*	F	GAGCCTGTGAGCGTGCTTTT	163	NM_001077840.1
	R	TGGTGCTGAGGATGACATGG		
*β-actin*	F	GCGGCATTCACGAAACTACCTT	268	NM_173979.3
	R	TCCTGCTTGCTGATCCACATCT		

Bcl-2, B-cell lymphoma-2; Bax, Bcl-2-associated X protein.
